# Isolated relapse of plasma cell leukemia in the central nervous systems: a case report and literature review

**DOI:** 10.1007/s12185-023-03545-7

**Published:** 2023-02-03

**Authors:** Takafumi Obo, Ken Morita, Yutaro Sumida, Kumi Nakazaki-Watadani, Masako Ikemura, Koichiro Yasaka, Osamu Abe, Hirokazu Takami, Shunsaku Takayanagi, Shota Tanaka, Hiroaki Maki, Yosuke Masamoto, Akiyoshi Miwa, Mineo Kurokawa

**Affiliations:** 1 Department of Hematology & Oncology, Graduate School of Medicine, The University of Tokyo, 7-3-1 Hongo, Bunkyo-Ku, Tokyo, 113-8655 Japan; 2 Department of Pathology, Graduate School of Medicine, The University of Tokyo, Tokyo, Japan; 3 Department of Radiology, Graduate School of Medicine, The University of Tokyo, Tokyo, Japan; 4 Department of Neurosurgery, Graduate School of Medicine, The University of Tokyo, Tokyo, Japan; 5 Department of Hematology, Tokyo-Kita Medical Center, Tokyo, Japan

**Keywords:** Plasma cell leukemia, Multiple myeloma, Extramedullary plasmacytoma, Central nervous system

## Abstract

Plasma cell leukemia is a rare yet aggressive form of multiple myeloma characterized by high levels of plasma cells circulating in the peripheral blood. We recently experienced a case of plasma cell leukemia that had been in stringent complete remission for nine years after autologous stem cell transplantations with subsequent courses of lenalidomide maintenance therapy, and then relapsed as an extramedullary plasmacytoma in the central nervous system. Assessment of the bone marrow did not prove proliferation of plasma cells at relapse, but imbalanced elevation of serum levels of free light chains was observed without changes in other clinical biomarkers including immunoglobulin levels. Salvage chemotherapy with isatuximab, pomalidomide, and dexamethasone (IsaPD) was promptly initiated. After two courses of IsaPD, significant remission was achieved and the neuronal symptoms completely resolved. When excessive serum levels of clonotypic free light chains are noted, their significance should be carefully assessed even when plasma cell propagation in the bone marrow is not observed. In such cases, hematologists should search for extramedullary proliferation of plasma cells, including in the immune-privileged central nervous system.

## Introduction

Plasma cell leukemia is a rare but aggressive entity of plasma cell dyscrasia. Plasma cell leukemia is defined by the presence of five percent or more circulating plasma cells in the peripheral blood smears in patients otherwise diagnosed with symptomatic multiple myeloma [1]. Approximately 1–2% of patients with multiple myeloma have plasma cell leukemia at diagnosis. The prognosis of primary plasma cell leukemia is generally dismal with reported overall survivals below one year. Despite the emergence of proteasome inhibitor-based induction regimens combined with autologous stem cell transplantations, the overall survival for the patients with plasma cell leukemia has only improved marginally, and its rate remains below 30% at four-year [2–4]. Relapse of plasma cell leukemia usually happens in the bone marrow, and relapse at extramedullary sites, especially in the central nervous systems is very rare. We have recently experienced a case of plasma cell leukemia that had been in a stringent complete remission for nine years, and then relapsed as an extramedullary plasma cell leukemia in the central nervous system. The bone marrow assessment at relapse resulted normo-cellular bone marrow without expansion of plasma cells. The only abnormality that we have detected was gradual elevation of the serum lambda free light chain levels. We speculate that the immune-privileged central nervous systems provided a sanctuary for the leukemia cells to escape courses of chemotherapy in the present case. When noted, imbalanced elevation of serum levels of the free light chains, even without propagation of plasma cells in the bone marrow, should be carefully assessed, and hematologists in practice are encouraged to search for extramedullary proliferation of plasma cells including the central nervous systems in such cases.

## Case

**Fig. 1:**
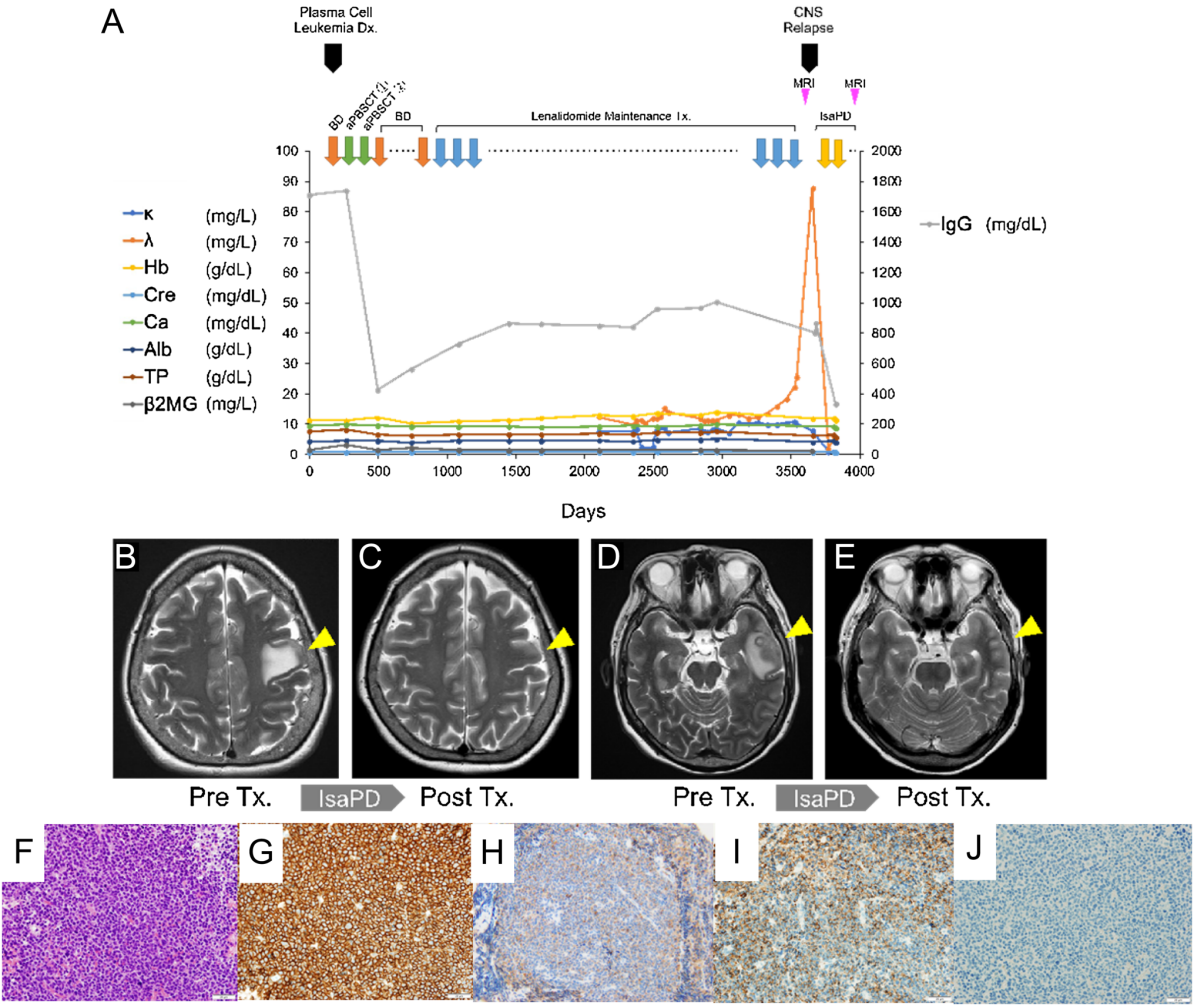
Isolated Relapse of Plasma Cell Leukemia in the Central Nervous System. **A** The clinical course of the patient. κ:serum levels of kappa-type free light chain, λ; serum levels of lambda-type free light chain, Hb; hemoglobin levels, Cre; serum levels of creatinine, Ca; serum levels of calcium, Alb; serum leveles of albumin, TP; serum levels of total protein, β2MG; serum levels of beta 2 microglobulin, IgG; immunoglobulin G, BD; bortezomib and dexamethasone therapy, aPBSCT; autologous peripheral blood stem cell transplantation, CNS; central nervous systems, IsaPD; Isatuximub, pomalidomide and dexamethasone therapy, MRI; magnetic resonance imaging, Dx; diagnosis. **B** and **D** Pre-treatment axial projections of the brain T2-weighted magnetic resonance imaging (MRI) at the relapsed phase showing infiltrating cerebral lesions in the parietal lobe (Panel B; arrow head) and in the temporal lobe (Panel D; arrow head). **C** and **E** Post-treatment axial projections of the brain T2-weighted MRI at the relapsed phase showing resolutions of the cerebral lesions in the parietal lobe (Panel C; arrow head) and in the temporal lobe (Panel E; arrow head). Images were taken after two cycles of the IsaPD regimen. **F**–**I** Morphologic examination and immunohistochemical stains performed on the cerebral biopsy. The Hematoxylin and Eosin (H&E)-stained section of the biopsy showed diffuse infiltration of plasma cells (Panel F) that are positive for CD138and IgG (Panel G and H). The plasma cells showed lambda light chain restriction [λ-in situ hybridization (ISH) (Panel I) and κ-ISH (Panel J)]. Scale bars; 20 μm. Magnifications; 40 x

A 68-year-old Japanese female was referred to our hospital for gradually worsening intracranial neuronal symptoms of confusion and expressive aphasia for the past two weeks. The patient had originally been diagnosed as monoclonal gammopathy of undetermined significance (MGUS) 18 years ago, and progressed to the plasma cell leukemia nine years ago. The patient had undergone induction chemotherapy with bortezomib and dexamethasone, followed by two autologous stem cell transplantations. During courses of the lenalidomide maintenance therapy, the patient had been in a stringent complete remission with the normal serum levels of free light chains. The systemic imaging studies resulted negative with the ^18^F-fluorodeoxyglucose Positron Emission Tomography (FDG-PET) and the ^11^C methionine Positron Emission Tomography (MET-PET) scans, which are the two most powerful methods to detect a minimal residual disease (MRD) in patients with plasma cell malignancies [5]. Nine years after the initiation of the lenalidomide maintenance therapy, gradual, yet significantly imbalanced elevation of the free light chains had appeared (Fig. 1A). Contrast-enhanced magnetic resonance imaging (MRI) was immediately taken, which showed hyperintense [T2-weighted and Fluid-Attenuated Inversion Recovery (FLAIR)] intracranial lesion in the parietal and temporal lobes with surrounding edema (Figs. 1B and D). Analysis of the cerebrospinal fluid revealed increased level of immunoglobulin G (IgG) with IgG index of 1.14. Result of the cerebrospinal fluid cytodiagnosis showed infiltration of aberrant plasma cells into the central nervous systems. The patient was admitted to the hospital, and the craniotomy brain biopsy was promptly performed. Histopathological assessment of the lesion proved propagation of the plasma cells with eccentric nuclei in the brain parenchyma, mainly infiltrating around the blood vessels (Figs. 1F–I). The aberrant proliferation of the plasma cells showed MIB1 positivity of 70%. Flow cytometry analysis confirmed clonal expansion of plasma cells that are positive for CD 138, CD 79a, CD 56, and λ-type light chain. These cells were negative for CD 3, CD 10, CD20, LCA, and κ-type light chain. The chromosomal analysis resulted normal karyotype (46XX) by G-banding, while the fluorescence in situ hybridization (FISH) analysis resulted positive for IGH-FGFR3 [t (4;14)]. Genetic analysis for the IgH clonality was also positive. These surface marker expression profiles and chromosomal translocations are shared between the myeloma cells at the initial diagnosis nine years ago and at the relapsed phase. To our surprise, assessment of the bone marrow aspiration did not prove propagation of plasma cells (0.6%). Complete blood counts revealed slightly decreased numbers of white blood cells (WBC) of 2800/μL and platelets of 1.07 × 10^5^/μL, with normal hemoglobin levels of 12.0 g/dL. The results of the serum biochemistry [Total protein (TP) 6.2 g/dL, Albumin 3.8 g/dL, Immunoglobulin G (IgG) 799 mg/dL] and the urinary analysis (TP/Creatinine (Cr) 77.9 mg/g x Cr) were insignificant, except for the imbalanced elevation of the serum free light chain levels (kappa = 7.7 mg/L, lambda = 87.5 mg/L, κ/λ = 0.088). As shown in the Fig. 1A, levels of the clonotypic free light chains had elevated gradually in the past three month before the onset of neuronal symptoms. Systemic work-up with computed tomography (CT) scan resulted negative for any other lesions than the cerebral tumor that was revealed by MRI. Collectively, the patient was diagnosed as the relapsed plasma cell leukemia in the form of extramedullary cranial plasmacytoma without involvement of the bone marrow. Combined chemotherapy with isatuximub, pomalidomide, and dexamethasone (IsaPD) was subsequently initiated. The brain radiotherapy was not chosen as the initial treatment for the disease due to potential development of late toxicities, such as cranial neuropathies and cognitive impairment. The neuronal symptoms rapidly improved after one course and enhanced MRI that was taken after two courses of IsaPd regimen showed significant reduction in size of the cerebral plasmacytoma (Figs. 1C and E). In addition, the IgG value in the cerebrospinal fluid decreased significantly almost to the normal level. The patient is currently on the fifth course of the IsaPD regimen without noticeable side effects.

## Discussion

Immunoglobulins are composed of heavy and light chains. Plasma cells make a small amount of extra light chains that do not bind with heavy chains, and these are called free light chains. In patients with plasma cell neoplasms, the multi-clonal production of light chains is overwhelmed by the monoclonal production by the transformed plasma cells, which subsequently affects the kappa to lambda ratio of the free light chains. Since the concept was originally introduced by Dr. Henry Bence Jones in 1847, the analysis of serum levels of the free light chains is now a standard as well as an essential examination in the diagnosis and management of plasma cell neoplasms [6]. Although the levels of serum free light chains can be affected by systemic inflammations such as infections or autoimmune diseases, it is still by far the most sensitive laboratory test to monitor the propagation of plasma cells in human bodies [7]. As in the present case, imbalanced elevation of serum levels of the free light chains could be the initial and the only clinical manifestation in an early phase of relapsed plasma cell neoplasms. When noted, it should be carefully assessed even without observation of plasma cells propagation in the bone marrow, and hematologists in practice are encouraged to search for extramedullary proliferation of plasma cells in such cases.

Reviewing the past literatures resulted only five cases of plasma cell leukemia that had relapsed in the central nervous systems after the initial treatment (Table 1). Notably, duration of remission after the initial treatment in our case is by far the longest (nine years) compared to the other four cases (1–28 months). This might be explained, but not fully, by the utilization of proteasome inhibitor-based induction chemotherapy and maintenance chemotherapy with immunomodulatory drugs (IMiDs) in our case. The striking fact from this table is the lack of bone marrow involvement at the time of extramedullary progression of the disease in any of the five cases. It is speculated that the central nervous system provided a sanctuary site for the malignant plasma cells to escape from courses of the chemotherapies, and these cells gradually grow in size until they begin to produce neuronal symptoms. Pomalidomide is a third-generation IMiDs, and it has been shown to exhibit an excellent central nervous system penetration and antitumor effect against plasma cell neoplasms [8–10]. The effectiveness of IsaPD therapy in the present case encourages us to consider IMiDs-based regimens in the relapsed cases of plasma cell leukemia with central nervous systems involvement. Generally, the Isatuximab is thought to be unable to pass the blood brain barrier. However, in certain circumstances including post-brain biopsies, the blood–brain barrier is partly compromised, which enables passive transition of the drug into the brain [11]. Thus, potential use of monoclonal antibodies should be considered in such cases.

**Table 1:** Previously reported cases of relapsed plasma cell leukemia in the central nervous systems

Ref	Age/sex	Ig. Type	Karyotype	Initial treatment	Maintenance therapy	Duration of remission	Relapsed sites	Treatment at relapse	Bone marrow involvement at relapse	Outcome
Our case	68 y/F	IgG-λ	46XX, t (4,14)	BD, autoPBSCT	Lenalidomide	9 y	Cerebrum	IsaPD	None	Alive, 5 m after relapse
[12]	61 y/M	IgA-λ	45–48, XY, + 1.del (1) (p22), add (7) (q36), del (8) (q22), − 10, − 16, + mar1(cp20)	VAD, autoPBSCT	None	2 y and 4 m	Lumber spinal cord	Surgery, Radiation, MTX, CyA, mPSL,	None	Dead, 1 y 9 m after relapse
[13]	44 y/F	IgG-κ	48, XX, + 3, i(7) (q10), dic (9;?) (q34;?), − 10, − 13, − 14, + 21, + ? 22, + 3 mar	VAD, alloBMT	None	1 y	Lumber spinal cord	Surgery, Radiation, VRB, VP-16, CPM, EDAP	None	Dead, 1 y after relapse
[13]	68 y/F	IgG-κ	46XX	VAD, autoPBSCT	None	7 m	Cerebellum	EDAP, MTX, CyA, Radiation	None	Dead, 9 m after relapse
[14]	54 y/M	λ type	N.A	PCAB,	None	1 m	Thoracic spinal cord, testis, cauda equina, lacrimal grand	MTX, Radiation, DT-PACE, alloBMT	None	Alive, 1 m after relapse

Clinical information of the five reported cases of plasma cell leukemia with CNS relapse is shown. Note that none of these cases had bone marrow involvement in the relapsed phase

*Ref* References, *F* female, *M* male, *VAD* vincristine, doxorubicin, and dexamethasone, *autoPBSCT* autologous peripheral blood stem cell transplant, *BD* bortezomib, dexamethasone, *IsaPD* isatuximab, pomalidomide, and dexamethasone, *Ig* immunoglobulin, *y* years, *m* months, *alloBMT* allogeneic bone marrow transplantation, *MTX* methotrexate, *CyA* Cytarabine, *mPSL* methylprednisolone, *EDAP* etoposide, dexamethasone, cytosine arabinoside and cisplatinum, *CPM* cyclophosphamide, *VRB* vinorelbine, *VP-16* etoposide, *PCAB* prednisone, cyclophosphamide, adriamycin and carmustine, *DT-PACE* dexamethasone, thalidomide, cisplatin, adriamycin, cyclophosphamide and etoposide, *N.A.* data not available

In summary, we have experienced a case of plasma cell leukemia that had been in a stringent complete remission for nine years after autologous stem cell transplantations with courses of lenalidomide maintenance therapy, and then relapsed as an extramedullary plasmacytoma in the central nervous system. Imbalanced elevation of serum levels of the free light chains could be the initial and the only clinical manifestation at the time of relapse, and thus systemic work-up including the central nervous systems would be necessary to examine the presence of extramedullary lesions especially when the bone marrow assessment resulted negative for the aberrant propagation of plasma cells.

## Data Availability

Relevant clinical data is available upon request.
